# Integrated twitter analysis to distinguish systems thinkers at various levels: a case study of COVID-19

**DOI:** 10.1007/s41109-022-00520-9

**Published:** 2023-02-17

**Authors:** Harun Pirim, Morteza Nagahi, Oumaima Larif, Mohammad Nagahisarchoghaei, Raed Jaradat

**Affiliations:** 1 Industrial and Manufacturing Engineering, North Dakota State University, Fargo, ND 58108 USA; 2 DiversityInc Media LLC, West Palm Beach, FL 33405 USA; 3 Industrial and Systems Engineering, Mississippi State University, Mississippi State, MS 39762 USA; 4 Computer Science and Engineering, Mississippi State University, Mississippi State, MS 39762 USA

**Keywords:** Systems thinking, Systems skills, COVID-19, Pandemic, Social networks, Twitter analysis, Follower network, Network clustering

## Abstract

Systems Thinking (ST) has become essential for practitioners and experts when dealing with turbulent and complex environments. Twitter medium harbors social capital including systems thinkers, however there are limited studies available in the extant literature that investigate how experts' systems thinking skills, if possible at all, can be revealed within Twitter analysis. This study aims to reveal systems thinking levels of experts from their Twitter accounts represented as a network. Unraveling of latent Twitter network clusters ensues the centrality analysis of their follower networks inferred in terms of systems thinking dimensions. COVID-19 emerges as a relevant case study to investigate the relationship between COVID-19 experts’ Twitter network and their systems thinking capabilities. A sample of 55 trusted expert Twitter accounts related to COVID-19 has been selected for the current study based on the lists from Forbes, Fortune, and Bustle. The Twitter network has been constructed based on the features extracted from their Twitter accounts. Community detection reveals three distinct groups of experts. In order to relate system thinking qualities to each group, systems thinking dimensions are matched with the follower network characteristics such as node-level metrics and centrality measures including degree, betweenness, closeness and Eigen centrality. Comparison of the 55 expert follower network characteristics elucidates three clusters with significant differences in centrality scores and node-level metrics. The clusters with a higher, medium, lower scores can be classified as Twitter accounts of Holistic thinkers, Middle thinkers, and Reductionist thinkers, respectfully. In conclusion, systems thinking capabilities are traced through unique network patterns in relation to the follower network characteristics associated with systems thinking dimensions.

## Introduction

Experts and practitioners need to address the increasing challenges of today’s sociotechnical systems while maintaining and elevating their performances under increasing complexities. These challenges include (1) requirement of high level systems integration to serve overarching goals, (2) lack of clarity to allow for decision support and commitment to alternative courses of action, (3) uncertainty caused by incomplete knowledge of systems and the unintended consequences, and (4) the effects of interdependence where there is mutual influence among systems and their related elements making analysis difficult (Boardman and Sauser 2006; Keating 2008; Nagahi et al. 2020a, b; Ullah Ibne Hossain et al. 2020). These four elements are likely to escalate as the interdisciplinary system problems of the twenty-first century continue to blur the lines between technical, social, managerial, and policy considerations (DeLaurentis and 2005, January. 2005; Gorod et al. 2008; Jaradat et al. 2017; Nagahi et al. 2020c; Karam et al. 2020). In response to these challenges, it is necessary to develop tools, pipelines and skills to support a more holistic, i.e. systemic, approach when dealing with complex system problems.

Sociotechnical failures disclose technical, sociopolitical, and power elements, or interactions between these elements (Nagahi et al. 2020a; Ullah Ibne Hossain et al. 2020; Frank 2006; Clegg 2000). A holistic frame of reference is necessary to provide effective solutions to address such rigorous failures. Consequently, studying the relationship between Systems Thinking (ST) skills and social media analytics, i.e. Twitter analysis, combines some social and technical aspects that might uncover information to support effective management of complex multidimensional systems. For example, the assessment of ST skills using social media analytics can help governments and the public distinguish the more accurate and systemic responses to the COVID-19 global pandemic to make informed decisions better in this critical and life-matter situation; given the contradictory statements, actions, and mismanagement of the pandemic at its early stage.

Although much has been written about systems thinking, the impact of systems thinking skills of COVID-19 experts on their tweets and Twitter follower networks to ensue is not elaborated.

This study aims to address the interdisciplinary literature gap in complex systems where ST skills are deciphered using social media analytics including Twitter and follower network analysis. Unraveling of latent Twitter network clusters is followed by the centrality analysis of the follower networks inferred in terms of systems thinking dimensions. A ST skills instrument (Jaradat 2015) is used to denote levels of systems thinking. The literature review identifies research gaps relevant to evaluating if experts' system thinking skills can be revealed through Twitter analysis. This article presents an overview of the relevant literature to integrate ST and social media analytics, followed by the data collection procedure, extraction of Twitter features for network construction and clustering, and follower network analysis. The results are analyzed and interpreted. Conclusions, limitations, and future work ensue.

## Review of literature

### Network analysis

Network representation encodes individuals as nodes, and relations between them as edges. The simple yet powerful representation encompasses information hidden under micro-to-macro scale network features. Microscale features emphasize certain node or edge characteristics, such as the most critical person or relationship. In contrast, macro-scale features reveal information about the network itself, such as the density of connections in the network. Mesoscale features existing in between provide distinct information, such as communities inside the network. Some examples of social networks include social media networks, business networks, and information circulation networks.

Several studies have been conducted using network analysis to tackle systems thinking-related topics. For instance, Dowd et al. (2018) proposed a new comprehensive decision-making framework for the maintenance, upgrading, and modernization of aging transportation infrastructure that includes highways, railways, bridges, and navigable waterways. This framework has three stages: (1) employ a systemic thinking approach to identify the impact factors, (2) complex network analysis to assess the criticality of the location of each component within the system, (3) apply a learning method to eliminate judgment and reduce the subjective disadvantages of learning algorithms currently available when using real data.

The results showed that this framework provides decision-makers with an index number representing the need for maintenance and modernization of each project and a hierarchical list in terms of essentiality. Tang et al. (2019) addressed the cascading effects in urban areas of earthquakes. The network analysis approach disclosed interrelated secondary events to generate ideas for designed strategies to mitigate disaster from systems thinking perspectives.

Network analysis is also applied in disaster risk management. In order to comprehend public security risks with associations, Tang and Lai (2019) suggested an approach to measure, analyze the public security risks, and provide an overview of effective mitigation strategies using social network analysis. The results showed that the framework added new value to the traditional risk management paradigm by offering support to urban managers developing comprehensive risk alleviation systems, hence, reducing risk interactions and their spread. Shiue et al. (2010) examined the social ties and perceived risk by using anonymity, offline activities, understanding quality, and media richness. This study showed that perceived risk and social ties were critical segments of social loafing. Social ties and perceived risk are hypothesized to affect social loafing within the online community.

Priven and Sacks (2015) adopted social network analysis to measure and analyze the communication levels between subcontractors. Pizzol and Scotti (2013) employed a systems thinking approach to identify marginal suppliers with geographical market delimitations where geographical markets are determined using network-based clustering. The results of this study showed that the proposed method could be applied to different products for which the trade network data are accessible, and it helps to provide a detailed analysis of technological constraints.

Health information available through social media has the potential to affect public health, especially when the population grows old. However, there is a lack of information about age-associated diseases on popular social media. Robillard et al. (2013) evaluated social media usage to share information about dementia, what sources of information about dementia were shared, and which dementia subjects commanded the conversation. The results showed that the dementia research community might benefit from the Twitter medium.

Health-related communities use social media frequently. Liu et al. (2016) investigated diabetes-related individuals on Twitter by depicting the frequency and timing of diabetes-related tweets, the geography of tweets, and the type of members over a two-year test of 10% of all tweets. The results showed that Twitter was becoming a space for online conversations about diabetes. Malik et al. (2014) evaluated the human advice-seeking behavior of primary health care (PHC) physicians. The study showed that SNA supported provider needs and presenting informal social interaction with physicians helped them use their professional linkages to seek advice since there was a lack of competent supervisory staff and a lack of improving functional indicators rather than clinical guidance. Blanchet et al. (2014) examined the sustainability indicators in rehabilitation sectors, contemplating differences in the governance and shape of the existing in two rehabilitation sectors. Differences in the structure of social networks marked greater after two years.

Lastly, in the tourism field, William et al. (2015) explored the content and structure of online word-of-mouth (eWOM) and its effect at a tourism destination when a festival was staged using both social network and text analysis. The results showed that Twitter users trusted seemingly disinterested opinions from individuals outside their social network.

### Twitter analysis

Twitter, standing as one of the most widely used social media, provides various data such as tweet and retweet statistics, demographics, relations to followers, hashtags for particular topics. Global information exchange favors Twitter to be a platform where users read and post messages known as 'tweets' connecting and interacting with various communities. Users share their daily life interests, post their opinions on timely subjects such as brands and places. This platform has been considered an important access point for information and data. For instance, Tsimonis and Dimitriadis (2013) evaluated why organizations created brand pages in social media, how they used them, and what approaches, policies, and procedures they followed based on collected data from Twitter and other social media platforms. The authors recognized different opportunities for organization managers recommending practices for powerful and effective social media handling. Using data collected from Twitter, Koo et al. (2011) explored how an employee could utilize social communication technologies to suit his/her challenge characteristics because of the growing significance of social communication technologies within an organization. Additionally, the worker’s social relationships were tested in terms of moderate media use in job surroundings and the way this use encouraged the undertaking performance of selecting telephone, video conferencing, email, immediate messaging, and blog was tested. The use of social technology resulted in positive challenge performance. Rui et al. (2012) conducted a study where they examined whether and how Twitter WOM affected film sales through a dynamic panel fact model using a public data and machine learning algorithm. The results of this study showed that the effect of WOM on product sales from Twitter was higher when users had more followers compared with users with fewer followers. Moreover, tracking and analyzing people’s sentiments and intentions on Twitter showed that negative WOM decreased product sales, whereas positive WOM increased them. Kontopoulos et al. (2013) proposed an authentic ontology-based approach towards a more efficient and powerful sentiment analysis of Twitter posts. The purpose of this approach was to analyze the received sentiment from each distinct motion in a post instead of characterizing the posts by a sentiment score that was used by machine learning-based classifiers. The Twitter analysis is also used in the political domain to provide important information about individuals` thoughts, beliefs, and expectations. Golbeck and Hansen (2014) proposed a new approach to estimate and compute the political preferences among an organization’s Twitter followers. Twitter analysis is also used to distinguish agriculture area. Bastos et al. (2018) used the generic versus specialized information shared on Twitter network topology to identify communities and associations matching the agricultural area. The results of this investigation showed that decentralization increases when the information shared was generic and that the network adopts centralization formations as conversations became more specialized. Lastly, Aharony (2010) conducted a study to explore the use of Twitter in academic and public libraries to comprehend microblogging patterns. The study showed that there were some contrasts between academic and public libraries, including the number of tweets, linguistic differences, and content.

## Methodology and data collection

### The research goal based on the research gap

**Fig. 1:**
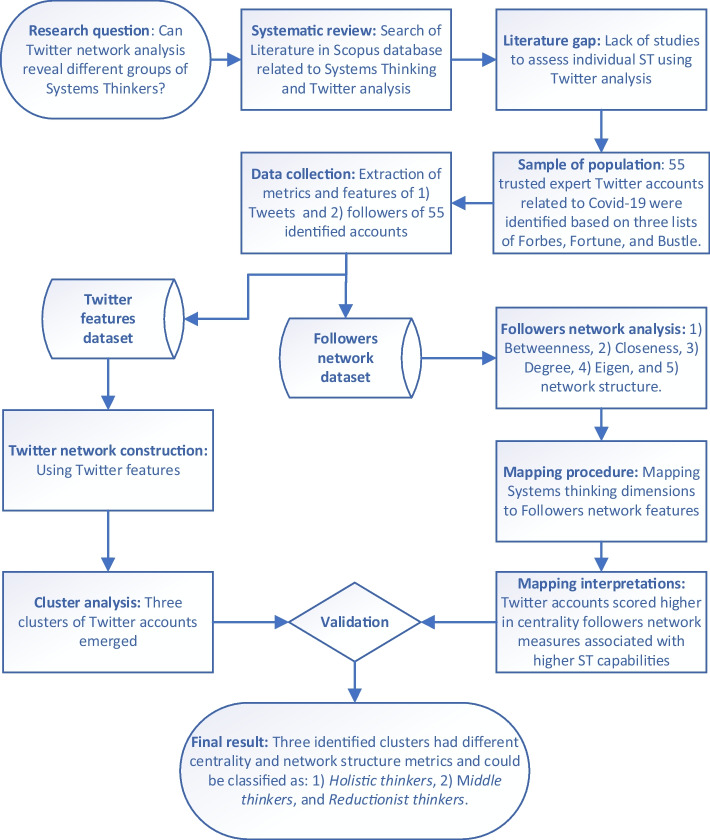
The research framework

As one of the most significant scientific sources globally, the Scopus database was used to systematically explore the literature. This systematic review's main goal was to find the articles that used Twitter analysis to assess levels of systems thinking capabilities of individuals or reveal unique network patterns observed for systems thinkers. As mentioned in the Table 4, a general search thread to find the relevant research articles in related subject areas, keywords, and journals published in the English language was conducted. Consequently, 235 articles were listed by the Scopus database. A careful review of these articles revealed that the literature lacks studies investigating the level of systems thinking of individuals in connection to their Twitter network profiles. Motivated by the research problem and the identified literature gap, a focused and critical case study was designed. The worldwide outbreak of the COVID-19 pandemic, a vital and complex problem, emerged as a potential use case to investigate the relationship between systems thinkers’ Twitter responses to complex problems and their systems thinking capabilities. The assessment of the systems thinking skills of trusted Twitter accounts related to COVID-19 tweets was based on the influence these Twitter accounts have on their worldwide followers and societies. The procedure of identifying these 55 Twitter accounts and corresponding data collections is introduced in the next sections. The research framework is presented in Fig. 1.

### Sample of population

In December 2019, the coronavirus (COVID-19) pandemic started to spread around the globe originally from China. In March 2020, the virus influenced almost most of the countries on every continent worldwide; consequently, the World Health Organization (WHO) declared the COVID-19 outbreak a worldwide pandemic. This virus is considered a danger to human health, resulting in worldwide fear and governmental quarantines. The side effect of this virus was somehow as dangerous as the virus itself for society. “As reported by the BBC, false information ranging from suggested medical care to conspiracy theories have been seen widely around the web, shared, and reposted by thousands of people on social media” (Wylde 2020); increased the number of casualties and pandemic fear. As a result of these threats, some credible media such as Forbes (Brown 2020), Fortune (Moore 2020), and Bustle (Wylde 2020) made up-to-date lists of trusted Twitter accounts of public health officials, researchers, epidemiologists, virus experts, family doctors, among others to assist in spreading accurate information, news, and recommendations to the masses to enhance society knowledge regarding COVID-19. The three trusted lists (i.e., Forbes, Fortune, and Bustle) were used in this research to identify credible organizations and individuals on Twitter as a sample of the current research population. A list of 55 Twitter accounts consists of 12 organizations such as WHO, GHS, and 43 individuals, have been selected for data collection. Table 5 presents the 55 Twitter accounts with information related to their account name, type of account, location, and the bio description on the corresponding Twitter account.

### Data collection procedure and twitter features dataset

To collect the data, we have applied a team developer access to Twitter through Application Programming Interface (API). After a few rounds of review, Twitter grants us team access to collect data. Twitter, by default, has some restrictions for tweets. The first restriction is it gives a maximum of 5000 tweets for each individual account. The second restriction is that we can access the information of 15 followers of each account every 15 min, which makes the follower analysis time-consuming and difficult. These two restrictions were the main limitations of data collection for the current research. R version 4.0 and “rtweet” library have been used to collect the data from Twitter. To start tweet collection, the “create_token” function using four API credentials from Twitter, including consumer key, secret consumer key, access token, and access secret, have been utilized, followed by the usage of “get_timeline” function to extract tweets of 55 identified Twitter accounts. First, the potentially important information from each Twitter account was extracted, such as the name of the account, id, screen name, location, description, follower counts, friends count, listed count, favorites count, status counts, and some other information. Then, all the tweets for each account were categorized into (1) organic tweets, (2) replies, and (3) retweets. The ratios for each class calculated for every Twitter account, as shown in Table 6.

### Follower analysis and features’ extraction

**Fig. 2:**
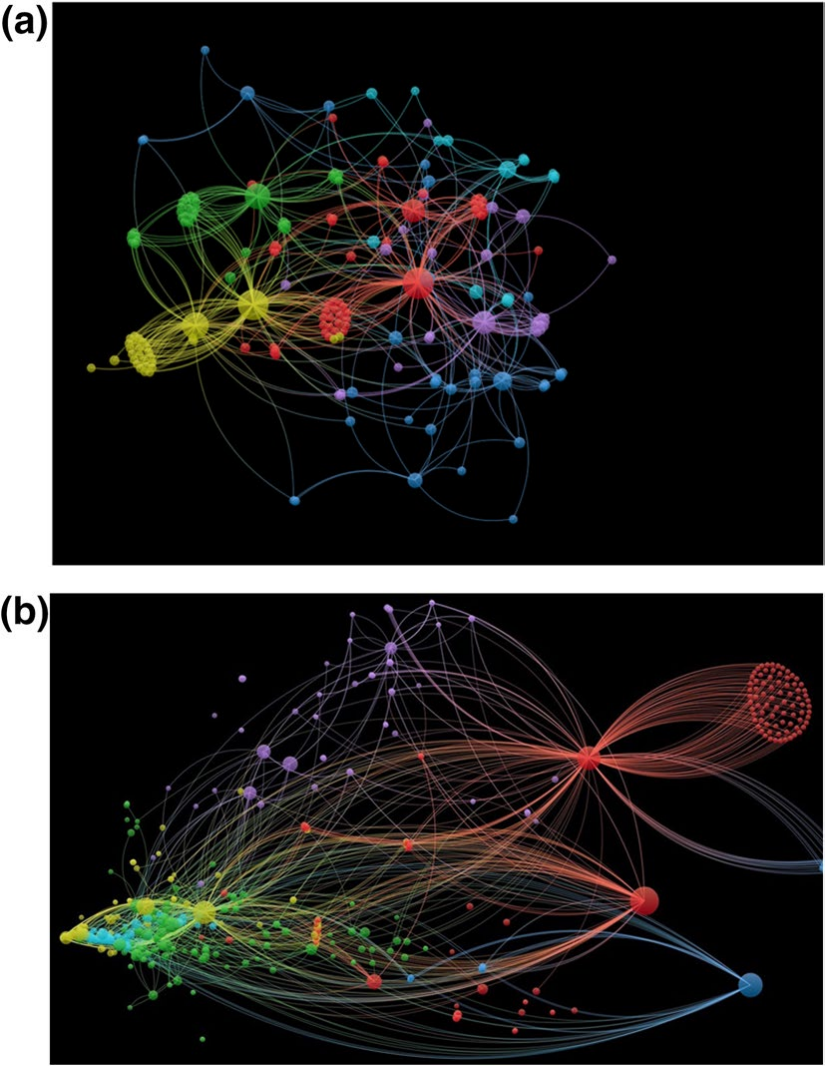
The followers’ network of Twitter account of **a** the University of Washington Virology and **b** World Health Organization (up to down)

R version 4.0 and rtweet, tidyverse libraries were used to collect and analyze the followers’ data of 55 identified Twitter accounts. The follower analysis was performed based on the modified code from Bellman (2018). Due to the main restriction of Twitter regarding followers’ data, only a sample of 45 followers is selected to perform the follower network analysis. To collect data from a sample of 45 followers of each account, 45 min needed, and a total of 55 × 45 min needed to collect the followers' network information for all identified Twitter accounts. After collecting the followers’ data for each Twitter account, we extracted some information regarding the number of followers, following, and friends of each account. Then followers’ networks were constructed to show the relationship between the identified Twitter accounts and their corresponding followers. Additionally, the number of nodes and edges in each followers’ network extracted after network construction. First, we constructed a general connection between Twitter users and their followers. Figure 2 depicts the followers’ network of the “University of Washington Virology (UWV)” and “World Health Organization (WHO)” Twitter accounts; the nodes represent Twitter followers, and edges represent the connection between Twitter accounts.

The two following networks are a one-step neighborhood network meaning only two layers of followers were considered. For example, the University of Washington Virology’s followers and followers of those followers were collected with the condition that followers of followers that were not connected with first-level followers of UWV were removed from the network.

Nodes were defined as users, and edges connected two users based on the “following relationship”. The intensity of a node was quantified by the centrality of that node.

VOSviewer software was used to cluster the network. VOSviewer’s clustering algorithm has two hyperparameters, such as the minimum size of a cluster, a threshold for the minimum size of a cluster, and a resolution parameter that helps handle the number of clusters. VOSviewer uses a local moving algorithm introduced by Van Eck et al. (2013) to solve this optimization problem (determining the distinct clusters). Figure 2, top and bottom, shows the UWV and WHO followers network consisting of 6 and 5 distinct clusters, respectively. Four centrality measures, namely, Betweenness centrality, Closeness centrality, Degree centrality, and Eigen Centrality, were chosen for followers’ network analysis, and the summary statistics for each measure (i.e., mean centrality, SD centrality, median centrality, min centrality, and max centrality) were generated as network features. The node-level metrics, such as “this account follows how many accounts,” “number of this account’s friends,” “number of nodes in the network of this account,” and “number of edges in the network of this account,” were added to the features of followers’ network.

## Result and discussion

### Twitter network construction

The Twitter network is constructed, making use of 104 features extracted from 55 Twitter accounts. These features are summarized in Table 7. The first 12 features belong to Twitter account metrics. Next 40 features reflect some statistics related to all tweets, (1) favorite count, (2) retweet count, (3) quoted_favorite_count, (4) quoted_retweet_count, (5) quoted_followers_count, (6) quoted_friends_count, (7) quoted_statuses_count, (8) retweet_favorite_count, (9) retweet_retweet_count, (10) retweet_followers_count, (11) retweet_friends_count, and (12) retweet_statuses_count. The next 25 features are relevant to some statistics of organic tweets (no replies and retweets) such as favorite count, retweet count, quoted_favorite_count, quoted_retweet_count, quoted_friends_count, and quoted_statuses_count. The next 6 features are obtained from the sources that the identified Twitter accounts generate the content, followed by 11 features as the top ten most frequently used words and the frequency of COVID-19 and its synonyms in the tweets of each Twitter account. Finally, ten features are related to the sentimental scores of the tweets associated with each Twitter account.

**Fig. 3:**
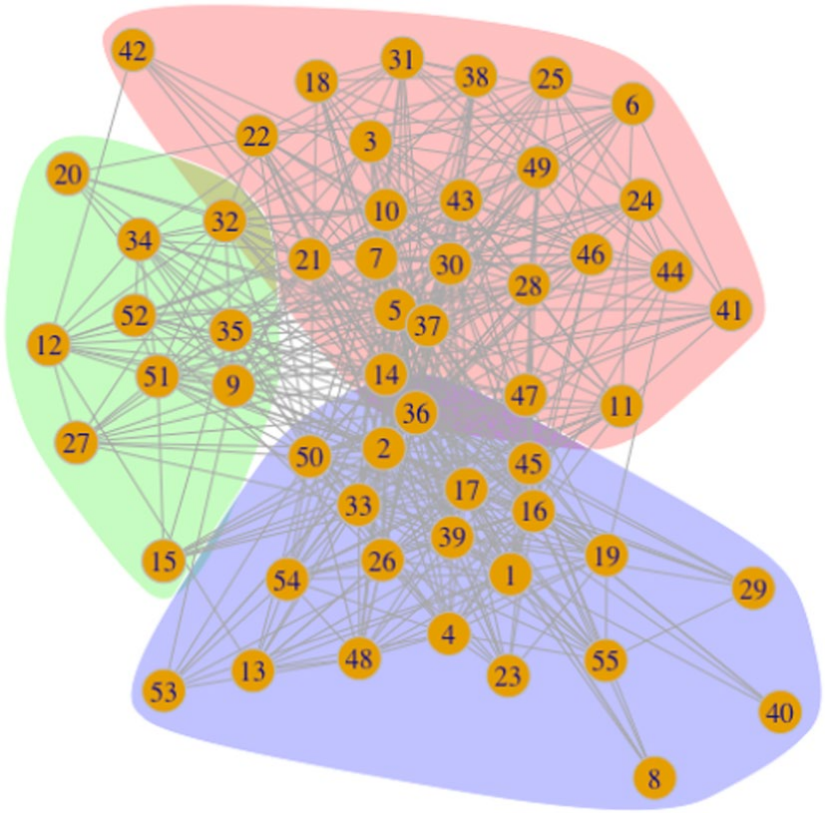
Three groups of systems thinkers revealed from the Twitter network: the purple group is enriched by holistic systems thinkers, the pink group is enriched by reductionist systems thinkers, and the green group is enriched by middle systems thinkers

Normalized Gower distance is used to quantify pairwise relationships among 55 accounts. Distance values are converted to a similarity matrix to construct the weighted network. Running a community structure finding (CSF) algorithm using the weighted network results in low modularity score (i.e. 0.072) detecting two clusters. CSF based on the modularity has resolution problem that it fails to find clusters at a finer resolution. In order to find clusters that result in better modularity of the network, the weighted network is binarized, retaining relationships stronger than 1.1 times the mean. Then, the community structure finding algorithm by Blondel et al. (2008) is applied to find similar Twitter accounts in the network. The algorithm detects three groups visualized in Fig. 3. These groups are further investigated to distinguish systems thinkers of various levels. It is critical to develop an objective strategy to map systems thinking qualities to some network measures. We made use of follower network features, including centrality scores and network-level metrics for mapping systems thinking dimensions to network measures.

### Follower analysis

Follower networks of 55 Twitter accounts, the followers’ features dataset are analyzed summarize statistics related to centrality and node-level metrics of each follower network (see Fig. 2) are extracted. Principle component analysis (PCA) is performed using follower data including betweenness, closeness, degree, eigen centralities, and node-level metrics. From five extracted features (that is, mean centrality, SD centrality, median centrality, min centrality, and max centrality) for betweenness centrality, one composite variable emerged. Additionally, one composite variable emerged for closeness and eigen centralities. Two composite variables emerged from degree centrality features. Table 1 indicates the summary of PCA for the centrality measures. To make all centrality composite variables the same scale, all the composite variables converted to percentage variables ranging from zero to 100 percent.

**Table 1:** PCA results of the centrality measures

Importance of components	Betweenness	Closeness	Degree		Eigen
Number of composite variables emerged	1	1	2	1	
Standard deviation (greater than 1 is preferred)	1.96	2.00	1.63	1.12	1.90
Proportion of variance explained (more than 60% is preferred)	77%	80%	53%	25%	90%

Moreover, the node-level metrics, such as out-degree (this account follows how many accounts), in-degree (number of this account’s friends), number of nodes in the network of this account, and number of edges in the network of this account were added to the five extracted composite centrality variables, as mentioned above. Finally, the five composite centrality variables and four node-level metrics were employed to interpret the followers’ characteristics of each of 55 Twitter accounts.

### Systems thinking mapping

In this study, the Systems thinking (ST) skills instrument developed by Jaradat (2015) is utilized to assess the level of systemic thinking of individuals in the domain of complex systems problems. The ST skills instrument is developed based on the principles of systems science and theory in the area of complex systems. The ST skills instrument has seven dimensions that measure preferences toward seven systemic skills needed to engage effectively in complex system problems, as shown in Table 2. The seven ST dimensions are mapped with the extracted measures from follower analysis to find any existence of potential connections between follower analysis and level of ST skills of individuals.

**Table 2:** The operational definition of the ST skills instrument (Jaradat et al. 2017)

Dimension	More systemic (holistic)
*Level of complexity*: Comfort with multidimensional problems and limited system understanding	*Complexity (C)* Expect uncertainty, work on multidimensional problems, prefer a working solution, and explore the surrounding environment
*Level of autonomy*: Balance between local-level autonomy versus system integration	*Integration (G)* Preserve global integration, tend more to a dependent decision and global performance
*Level of interaction*: Interconnectedness in coordination and communication among multiple systems	*Interconnectivity (I)* Inclined to global interactions, follow a general plan, work within a team, and interested less in identifiable cause-effect relationships
*Level of change*: Comfort with rapidly shifting systems and situations	*Tolerant of change (Y)* Prefer taking multiple perspectives into consideration, underspecify requirements, focus more on external forces, like long-range plans, keep options open, and work best in a changing environment
*Level of uncertainty*: Acceptance of unpredictable situations with limited control	*Emergence (E)* React to situations as they occur, focus on the whole, comfortable with uncertainty, believe the work environment is difficult to control, enjoy subjectivity and non-technical problems
*Level of hierarchical view*: Understanding system behavior at the whole versus part level	*Holism (H)* Focus on the whole, interested more in the big picture, interested in concepts and abstract meaning of ideas
*Level of flexibility*: Accommodation of change or modifications in systems or approach	*Flexibility (F)* Accommodating to change, like a flexible plan, open to new ideas, and unmotivated by routine

The eigen centrality is an ideal ‘all-round’ social network analysis score, well suited for comprehending human social networks (Bhasin 2019; Bonacich 1972; Disney 2020; Franceschet 2018) that calculates the impact of a node (here, a Twitter account) using its degree and the degrees of its neighbors.

Eigen centrality has a hypothetical positive correlation with all seven ST skills dimensions. For example, the Twitter account that scores high eigen centrality is well-connected with its followers and has well-linked followers; the individual (who owns the Twitter account) might have a high level of Interaction and Autonomy skills based on the operational definitions in Table 2.

The account with a high score in the eigen centrality influences the entire network effectively and proficiently with a good amount of information and knowledge it has. Thus, the individual owner of Twitter might possess good systems thinking skills such as level of Hierarchical View and Complexity. Finally, the account with a high Eigen score is more responsive to explaining and solving unexpected and emergent social problems discussed in the Twitter network. Therefore, the Twitter account owner might have high systemic skills regarding the level of Change, Uncertainty, and Flexibility. Overall, the eigen centrality measure can be a good representative of the level of systemic thinking skills of the Twitter account owner.

The degree centrality score is similar to the eigen centrality score in the sense that measures consider the well-connectivity of a node in a network system (Bhasin 2019; Disney 2020; European Molecular Biology Laboratory 2020; Freeman 1978; Levorato 2014). Interestingly, two components emerged from our follower network PCA results, which can be related to in-degree and out-degree measures. The two degree-centrality measures are powerful in identifying the connected and popular people in the network system who probably have good amounts of knowledge and information; they can also quickly link with the wider network system. The in-degree measure can be related to levels of Hierarchical View, Complexity, and Interaction. out-degree can be related to levels of Autonomy, Change, Uncertainty, and Flexibility. Thus, the Degree Centrality might be a good indicator of an individual's level of systems thinking skills in a network of Twitter.

The closeness centrality assigns a score to a node according to its “closeness” to all other nodes (i.e., the shortest paths exist among all the nodes of a network) in the network system (Disney 2020; European Molecular Biology Laboratory 2020; Sabidussi 1966). closeness centrality can identify the best-placed people to affect the whole network system in the fastest time. This measure can distinguish the influencers and “broadcasters” who can exchange information and knowledge effectively and quickly. There are many similarities between the features of closeness centrality and the dimensions of ST skills. People with a high closeness score might have levels of Interaction, Change, Uncertainty, Hierarchical View, and Flexibility.

The betweenness centrality computes the frequency of a node poses on the shortest path among other nodes of the network (i.e., bridging between network’s nodes) (Disney 2020; European Molecular Biology Laboratory 2020; Levorato 2014; Freeman 1977; Borgatti 2005; Morselli and Roy 2008). This measure is useful to find how influential a person is on the flow around a network system. This measure is a good indicator of communication dynamics in a network. People with high betweenness scores tend to control information and hold authority in a network system (i.e., a gatekeeper in a network), and removing these individuals from the network system might disrupt communication, dynamics, and flow in the network system. According to the definition of the betweenness centrality measure and Table 2, there are hypothetical connections between the betweenness centrality measure and different dimensions of the ST instrument. For example, there is a connection between the level of interaction (that is, “interconnectedness in coordination and communication among multiple systems”) and the level of Hierarchical View (that is, “understanding system behavior at the whole versus part level”) and Betweenness centrality measure.

Based on foregoing discussion, the four measures of centrality can serve as good indicators for assessing the level of seven ST skills for Twitter accounts with respect to the analysis of their followers’ network, as depicted in Fig. 2.

### Network clustering

Twitter features dataset (introduced in Sect.  4.A.) has been utilized to cluster 55 Twitter accounts. Since clustering is an unsupervised learning method that needs validation, the follower analysis results (introduced in Sect. 4.B.) are used to elucidate clusters. The emerged clusters of accounts shown in Fig. 3 are enriched based on account metrics pertaining to follower analysis. Table 3 shows the mean and SD of the Twitter analysis metric for each of the three clusters. Table 8 presents the details for all Twitter accounts grouped into three clusters. Tables 9 and 10 indicate the results of between-group ANOVA and Tukey HSD t-tests performed as post-hoc multiple-group comparison tests.

**Table 3:** The average and SD of Twitter analysis metrics for each of the three emerged clusters

Statistics	M	SD	M	SD	M	SD
Betweenness centrality	59.1%	30.1%	80.3%	15.0%	81.8%	16.6%
Closeness centrality	7.4%	8.2%	14.7%	11.8%	25.3%	29.9%
Degree centrality 1	64.3%	10.0%	72.7%	7.6%	72.1%	21.0%
Degree centrality 2	8.2%	4.1%	8.0%	7.5%	16.1%	22.3%
Eigen centrality	14.8%	7.94%	21.9%	14.9%	33.0%	27.8%
# Of following accounts	1472.7	1211.5	696.3	577.6	1761.7	1673.2
# Of friends’ accounts	162.8	453.2	3.2	3.4	9.1	17.9
# Of network nodes	855.4	529.8	459.1	311	284.5	211.6
# Of network edges	2110	1348	1111	765	691	527

Interestingly, Twitter accounts in cluster 3 have relatively higher follower network centrality measures than the other two clusters. Additionally, Twitter accounts in cluster 1 have relatively lower follower network centrality metrics than those in cluster 2. According to mapping the relationships between ST dimensions and Twitter accounts' centrality measures, we infer high scores in centrality measures of follower analysis associated with high ST capability of individuals. As a result, we conclude since Twitter accounts in cluster 3 have relatively higher centrality measures, the individuals corresponding to these Twitter accounts might have higher ST capability skills than others. Consequently, we call Twitter accounts in cluster 3 holistic thinker cluster. Similarly, the Twitter accounts in cluster 1 are reductionist thinkers due to low scores pertaining to their follower network analysis's centrality scores. Since Twitter accounts in cluster 2 have centrality scores almost between clusters 1 and 3, they are called middle thinker Twitter accounts. Results indicate the follower network analysis might be able to quantify systemic thinking capabilities of individuals based on their tweets in the case of the COVID-19 pandemic.

## Conclusion

Twitter is a crucial medium in circulating information, and unfortunately, sometimes erroneous or inadequate messages are propagated in a network by users. For example, in the case of the worldwide outbreak of the COVID-19 pandemic, we observed many unsupported claims about the origin of the virus, virus transmission, possible prevention, and likely treatment. In this confusing situation, knowing reliable sources and the level of holistic systems thinking of these sources greatly assist the public in making correct decisions. Thus, COVID-19 stands as a potential use case to investigate the relationship between COVID-19 experts’ Twitter network and their systems thinking capabilities.

The interdisciplinary literature review revealed that there was a literature gap connecting COVID-19 experts’ system thinking level and their Twitter network profiles. Therefore, we identified 55 trusted expert Twitter accounts based on the lists of Forbes, Fortune, and Bustle.

We constructed the Twitter network of these experts based on features extracted from their Twitter accounts, including organic metrics (e.g., account favorite count, list count, followers count, etc.), tweets’ measures (e.g., favorite count, retweet count, reply count, etc.), sentimental analysis, source of tweets as well as their tweets like most frequent words and their frequencies and frequency of using COVID-19 synonyms. Clustering the network based on tweets and Twitter features identified three distinct groups of experts emerged. The follower networks of experts were generated to elucidate clusters. The system thinking dimensions were mapped to follower network measures such as betweenness, closeness, degree, eigen centralities, and node-level metrics. By comparing the follower network measures of 55 experts, we inferred that three identified clusters had meaningful differences in centrality scores and node-level metrics. The cluster with a higher, medium, lower score can be classified as Holistic thinkers, Middle thinkers, and Reductionist thinkers, respectively.

Briefly, this research tested that the capabilities of individuals as system thinkers can reveal unique network patterns and distinct communities associated with the level of system thinking of COVID-19 experts.

Twitter restrictions related to the search of the past tweets and access to followers’ information of each Twitter account was one of the limitations of the study. Another limitation was the validity of mapping systems thinking dimensions to centrality measures of follower network analysis; further investigation with other samples of the population is needed to validate the current mapping. Future studies will be directed into investigating the relationship between Twitter users’ systems thinking and their sentiment analysis. Analyzing the organic tweets of expert Twitter accounts related to COVID-19 using text mining and natural language processing to better understand the impact of tweets on followers and spread knowledge and information within the Twitter network. The new research finding can be compared and combined with the current research results to analyze these studies' validity and consistency.

The results of the current study have some practical implications as follows:

- Using follower analysis and Twitter analysis to understand systems thinking of people, especially the influencers and celebrities, and how their role is important to spread the true news and knowledge to the community.
- How systems thinking is related to having a better network of followers promotes the more efficient and effective transformation of information and knowledge to the community.
- As an individual’s level of systems thinking skills can be enhanced, the social media activity of the individuals can be improved. This is very important since the research shows there is a necessity to create a safe and healthy virtual environment for everybody, so everyone can express their opinions and beliefs in the direction of the community’s values.

## Data Availability

The datasets used and/or analysed during the current study are available from the corresponding author on reasonable request.

## Appendix

See Tables 4, 5, 6, 7, 8, 9 and 10.

**Table 4:** The search thread for systematic literature search

Search criteria	Search thread
Search words	(twitter analysis) AND ((systems thinking)) AND (tweet)
Exact keywords (limited to)	"Social Media" OR "Social Networking (online)" OR "Twitter" OR "Human" OR "Decision Making" OR "Behavioral Research" OR "Social Network" OR "Sentiment Analysis" OR "Social Networks" OR "Social Network Analysis."
Document type (limited to)	Journal article
Language (limited to)	English
Subject area (limited to)	Engineering

**Table 5:** Selected twitter accounts of trusted officials and individuals related to COVID-19

	Account name	Org. versus Ind	Location
1	Global Health Strategies	Organization	NY/Delhi/Rio/Beijing/Nairobi/Joburg/London
2	Hannah Ritchie	Individual	Oxford, England
3	Adam Kucharski	Individual	London, England
4	Dr. Tara C. Smith	Individual	Kent, Ohio, USA
5	Dr Alexandra Phelan	Individual	DC, NYC, USA
6	Amesh Adalja	Individual	Pittsburgh, Baltimore, NYC, USA
7	Ashish "I'm still focused on testing" Jha	Individual	Cambridge, MA
8	Atul Gawande	Individual	Newton, MA, USA
9	Bill Hanage	Individual	London, England
10	Isaac Bogoch	Individual	Toronto, Canada
11	Timothy Caulfield	Individual	Edmonton, Canada
12	Dr. Robert R. Redfield	Individual	Atlanta, GA, USA
13	Celine Gounder, MD, ScM, FIDSA	Individual	New York, NY, USA
14	Steffanie Strathdee, PhD	Individual	San Diego, CA, USA
15	Caitlin Rivers, PhD	Individual	Baltimore, MD, USA
16	CNN Breaking News	Organization	Everywhere
17	David Juurlink	Individual	Toronto, Canada
18	Devi Sridhar	Individual	Edin, Oxford, England, Wash DC, Miami, USA
19	Drew A. Harris, DPM, MPH	Individual	Philadelphia, PA, USA
20	Mike Ryan	Individual	Geneva, Switzerland
21	Dr. Nancy Messonnier	Individual	Atlanta, GA, USA
22	Dr. Philippe Chouinard	Individual	Dieppe, New Brunswick, Canada
23	Tedros Adhanom Ghebreyesus	Individual	Geneva, Switzerland
24	Dr. Tom Frieden	Individual	New York, NY, USA
25	Dan Epstein	Individual	Washington, USA/ Geneva, Switzerland
26	Florian Krammer	Individual	New York, NY, USA
27	Helen Branswell	Individual	Boston, MA, USA
28	JAMA	Organization	Chicago, IL, USA
29	Jeremy TEST/TRACE/ISOLATE Konyndyk	Individual	Washington DC, USA
30	Johns Hopkins Bloomberg School of Public Health	Organization	Baltimore, MD, USA
31	Julia Belluz	Individual	Vienna, Austria
32	Kai Kupferschmidt	Individual	Berlin, Germany
33	Prof Palpatine, PhD	Individual	Brisbane, Australia
34	Max Roser	Individual	Oxford, England
35	Michael Mina	Individual	Boston, MA, USA
36	Marc Lipsitch	Individual	Boston, MA, USA
37	Dr Muge Cevik	Individual	Scotland, UK, EU
38	Maria Van Kerkhove	Individual	Geneva, Switzerland
39	The New York Times	Organization	New York City, USA
40	Doctor Radio	Organization	New York, USA
41	Vincent Racaniello	Individual	New York, USA
42	Project HOPE	Organization	Everywhere
43	APHA	Organization	Washington DC, USA
44	Roopa Dhatt	Individual	Washington, DC, USA
45	Save the Children US	Organization	Fairfield, CT, USA
46	Dr Sylvie Briand	Individual	Geneva, Switzerland
47	Scott Gottlieb, MD	Individual	Washington, DC, USA
48	Dr. Stephen M. Hahn	Individual	Silver Spring, MD, USA
49	Tom Inglesby	Individual	Baltimore, MD, USA
50	Trisha Greenhalgh	Individual	Oxford, England
51	Trevor Bedford	Individual	Seattle, WA, USA
52	U.S. FDA	Organization	Silver Spring, MD, USA
53	UW Virology	Organization	Seattle, WA, USA
54	Vivek Murthy	Individual	Washington, DC, USA
55	World Health Organization (WHO)	Organization	Geneva, Switzerland

**Table 6:** Percentage of organic tweets, retweets, and replies

#	Account name	Organic tweets %	Retweets %	Replies %
1	Global Health Strategies	61.3	28.8	9.9
2	Hannah Ritchie	23.8	27.4	48.8
3	Adam Kucharski	37.7	34.6	27.7
4	Dr. Tara C. Smith	10.4	23.1	66.6
5	Dr Alexandra Phelan	38.6	32.3	29.2
6	Amesh Adalja	90.9	8.0	1.1
7	Ashish "I'm still focused on testing" Jha	32.2	43.4	24.3
8	Atul Gawande	50.7	38.4	10.9
9	Bill Hanage	30.0	21.0	49.0
10	Isaac Bogoch	21.2	16.8	62.0
11	Timothy Caulfield	44.2	29.6	26.2
12	Dr. Robert R. Redfield	57.3	30.3	12.4
13	Céline Gounder, MD, ScM, FIDSA	17.4	61.5	21.0
14	Steffanie Strathdee, PhD	38.8	17.2	44.1
15	Caitlin Rivers, PhD	28.6	42.2	29.2
16	CNN Breaking News	83.4	16.3	0.2
17	David Juurlink	32.0	15.4	52.6
18	Devi Sridhar	57.4	25.0	17.6
19	Drew A. Harris, DPM, MPH	82.6	3.9	13.5
20	Mike Ryan	22.2	77.3	0.5
21	Dr. Nancy Messonnier	45.6	52.7	1.7
22	Dr. Philippe Chouinard	39.9	35.7	24.4
23	Tedros Adhanom Ghebreyesus	47.7	32.2	20.1
24	Dr. Tom Frieden	86.4	10.9	2.7
25	Dan Epstein	49.0	48.7	2.3
26	Florian Krammer	14.0	51.2	34.8
27	Helen Branswell	37.4	9.3	53.3
28	JAMA	46.3	47.8	5.9
29	Jeremy TEST/TRACE/ISOLATE Konyndyk	19.1	49.6	31.3
30	Johns Hopkins Bloomberg School of Public Health	59.4	38.6	2.0
31	Julia Belluz	32.6	45.5	21.9
32	Kai Kupferschmidt	18.5	27.7	53.8
33	Prof Palpatine, PhD	13.0	17.1	69.9
34	Max Roser	22.2	23.3	54.5
35	Michael Mina	10.8	11.5	77.8
36	Marc Lipsitch	29.2	37.9	32.9
37	Dr Muge Cevik	19.4	47.4	33.2
38	Maria Van Kerkhove	31.3	57.9	10.8
39	The New York Times	69.2	16.3	14.5
40	Doctor Radio	91.0	5.4	3.6
41	Vincent Racaniello	81.2	13.2	5.7
42	Project HOPE	72.2	22.3	5.5
43	APHA	45.9	49.3	4.8
44	Roopa Dhatt	24.0	63.0	13.0
45	Save the Children US	56.4	25.8	17.8
46	Dr Sylvie Briand	38.4	61.2	0.4
47	Scott Gottlieb, MD	27.8	52.7	19.5
48	Dr. Stephen M. Hahn	27.7	7.2	65.2
49	Tom Inglesby	42.8	37.9	19.3
50	Trisha Greenhalgh	23.2	24.0	52.7
51	Trevor Bedford	23.1	12.0	64.9
52	U.S. FDA	44.4	46.1	9.5
53	UW Virology	74.8	18.6	6.5
54	Vivek Murthy	61.2	20.7	18.1
55	World Health Organization (WHO)	6.5	31.1	62.4

**Table 7:** Extracted Twitter features

Main categories of Twitter features	Selected Twitter features
Twitter account metrics	followers_count
	friends_count
	listed_count
	favoritess_count
	statuses_count
	# of all tweets
	# of Organic tweets
	# of Retweets
	# of Replies
	% Organic tweets
	% Retweets
	% Replies
All tweets (including retweets and replies)	Favorite count
	Ave top 10
	SD top 10
	Median
	Mean
	Max
	Retweet count
	Ave top 10
	SD top 10
	Median
	Mean
	Max
	quoted_favorite_count
	Median
	Mean
	Max
	quoted_retweet_count
	Median
	Mean
	Max
	quoted_followers_count
	Median
	Mean
	Max
	quoted_friends_count
	Median
	Mean
	Max
	quoted_statuses_count
	Median
	Mean
	Max
	retweet_favorite_count
	Median
	Mean
	Max
	retweet_retweet_count
	Median
	Mean
	Max
	retweet_followers_count
	Median
	Mean
	Max
	retweet_friends_count
	Median
	Mean
	Max
	retweet_statuses_count
	Median
	Mean
	Max
Organic tweets (no retweets and replies)	Favorite
	Ave top 10
	SD top 10
	Median
	Mean
	Max
	Retweet
	Ave top 10
	SD top 10
	Median
	Mean
	Max
	quoted_favorite_count
	Median
	Mean
	Max
	quoted_retweet_count
	Median
	Mean
	Max
	quoted_followers_count
	Median
	Mean
	Max
	quoted_friends_count
	Median
	Mean
	Max
	quoted_statuses_count
	Median
	Mean
	Max
Source	TweetDeck
	Android
	iPhone
	web app
	Web Client
	Others
Most frequent words in tweets	Freq_w1
	Freq_w2
	Freq_w3
	Freq_w4
	Freq_w5
	Freq_w6
	Freq_w7
	Freq_w8
	Freq_w9
	Freq_w10
	Freq_ COVID-19_synonyms
Sentimental analysis	Anger
	Anticipation
	Disgust
	Fear
	Joy
	Sadness
	Surprise
	Trust
	Negative
	Positive

**Table 8:** Twitter analysis metric for all the Twitter accounts grouped into three emerged clusters

Cluster	Id #	Betweenness centrality (%)	Closeness centrality (%)	Degree centrality 1 (%)	Degree centrality 2 (%)	Eigen Centrality (%)	# of following accounts	# of friends’ accounts	# of network nodes	# of network edges
Cluster 1 (n = 24) Orange Circles	3	27.0	1.7	63.6	8.7	9.7	847	2	1232	3015
	5	70.0	5.5	67.0	7.7	16.1	3730	87	661	1632
	6	71.0	6.8	68.0	6.2	13.9	1961	18	616	1452
	7	53.0	2.3	57.5	13.6	12.9	443	1	1130	3076
	10	76.0	9.0	73.8	3.9	18.6	669	18	450	1028
	11	85.0	11.2	72.3	4.3	17.4	2501	78	420	961
	14	86.0	15.9	77.7	2.7	28.2	2796	2204	270	621
	18	69.00	5.60	66.0	7.4	10.9	385	0	713	1704
	21	56.00	3.30	49.9	15.3	8.30	855	3	1127	2997
	22	1.00	0.70	50.9	10.8	5.10	669	374	1974	4540
	24	9.00	1.40	42.0	15.3	5.8	257	5	1834	4534
	25	33.00	2.60	53.7	12.3	7.2	632	321	1254	3144
	28	97.00	40.3	79.4	3.30	38.6	812	1	123	293
	30	83.00	5.90	61.6	9.90	9.1	2512	5	581	1454
	31	0.00	0.00	50.1	15.2	6.9	2712	13	1836	4888
	37	87.00	12.9	79.2	1.60	27.8	1652	8	295	659
	38	59.00	3.70	62.2	8.00	11.7	481	9	959	2268
	41	90.00	12.1	71.2	4.00	18.4	354	0	424	962
	42	68.00	5.80	69.4	7.00	18.8	1293	412	627	1523
	43	72.00	8.10	70.2	6.10	17.9	4944	56	516	1248
	44	9.00	1.40	55.3	12.5	11.5	2163	269	1556	3950
	46	82.00	8.70	68.8	6.00	16.8	546	16	535	1253
	47	83.00	8.30	68.2	8.50	9.10	899	0	486	1237
	49	52.00	3.50	65.6	7.50	14.3	1231	7	910	2201
Cluster 2 (n = 10) Blue Circles	9	85.00	16.3	76.1	3.70	22.7	342	4	282	660
	12	90.00	12.2	81.1	28.0	20.6	484	1	392	923
	15	95.00	13.8	77.8	3.80	9.20	437	1	177	423
	20	62.00	4.50	62.9	8.40	6.70	205	1	830	1994
	27	90.00	21.9	78.1	3.10	36.2	1826	3	208	488
	32	65.00	3.60	62.4	9.10	12.5	1435	10	948	2309
	34	96.00	39.1	80.1	2.50	52.2	1102	8	145	344
	35	63.00	5.40	65.9	8.00	13.8	212	4	716	1742
	51	63.00	3.30	65.5	8.80	9.90	769	0	721	1805
	52	94.00	27.2	77.3	4.60	35.6	151	0	172	420
Cluster 3 (n = 21) Green Circles	1	72.00	4.60	67.0	7.70	16.0	2718	13	708	1728
	2	69.00	7.10	74.9	4.10	21.8	698	28	486	1134
	4	54.00	3.50	62.9	11.9	10.5	4634	17	845	2294
	8	91.00	13.6	84.6	26.9	30.8	434	2	311	738
	13	42.00	3.00	55.5	10.6	6.9	560	26	1155	2760
	16	100.00	100	96.2	20.4	100	120	0	52	109
	17	86.00	15.9	78.6	0.00	16.2	828	21	318	675
	19	65.00	1.40	0.00	46.5	0.0	17	20	893	3557
	23	95.00	22.3	76.5	5.40	38.8	890	0	161	404
	26	88.00	18.6	81.4	0.30	32.7	816	1	217	476
	29	58.00	2.70	50.5	14.5	10.7	521	5	1215	3138
	33	88.00	13.6	75.4	2.80	14.3	4511	15	344	778
	36	98.00	54.8	79.0	4.60	65.9	1526	0	89	223
	39	99.00	85.5	100	100	82.4	906	0	62	157
	40	99.00	84.9	98.6	17.9	84.8	1115	58	58	113
	45	75.00	8.80	70.1	5.60	16.2	4866	2	508	1200
	48	74.00	7.00	60.3	12.1	16.4	720	5	652	1728
	50	93.00	15.8	82.9	28.6	33.9	2078	9	289	708
	53	93.00	22.9	71.3	10.5	40.0	122	0	195	545
	54	83.00	10.1	70.1	3.80	15.9	53	2	503	1113
	55	95.00	35.3	78.2	3.7	38.5	1720	0	145	345

**Table 9:** The summary results of the post-hoc between-groups ANOVA test

Network measures	F	Sig
Betweenness centrality	6.124	.004
Closeness centrality	4.542	.015
Degree centrality 1	1.939	.154
Degree centrality 2	1.959	.151
Eigen Centrality	5.111	.009
This account follows	1.463	.241
Friends	1.778	.179
Node	6.028	.004
Edge	4.959	.011

**Table 10:** The results of post-hoc Tukey HSD tests

Dependent variable	(I) Cluster	(J) Cluster	Mean difference (I-J)	Std. error	Sig	95% Confidence interval	
						Lower bound	Upper bound
*Multiple comparisons (Tukey HSD)*							
Betweenness centrality	1.00	2.00	− .21217*	.08794	.050	− .4243	.0000
		3.00	− .22679*	.06981	.006	− .3952	− .0584
	2.00	1.00	.21217*	.08794	.050	.0000	.4243
		3.00	− .01462	.08976	.985	− .2312	.2019
	3.00	1.00	.22679*	.06981	.006	.0584	.3952
		2.00	.01462	.08976	.985	− .2019	.2312
Closeness centrality	1.00	2.00	− .07368	.07507	.592	− .2548	.1074
		3.00	− .17942*	.05960	.011	− .3232	− .0356
	2.00	1.00	.07368	.07507	.592	− .1074	.2548
		3.00	− .10575	.07663	.359	− .2906	.0791
	3.00	1.00	.17942*	.05960	.011	.0356	.3232
		2.00	.10575	.07663	.359	− .0791	.2906
Degree centrality 1	1.00	2.00	− .08403	.05619	.301	− .2196	.0515
		3.00	− .07779	.04461	.199	− .1854	.0298
	2.00	1.00	.08403	.05619	.301	− .0515	.2196
		3.00	.00625	.05736	.993	− .1321	.1446
	3.00	1.00	.07779	.04461	.199	− .0298	.1854
		2.00	− .00625	.05736	.993	− .1446	.1321
Degree centrality 2	1.00	2.00	.00242	.05427	.999	− .1285	.1334
		3.00	− .07849	.04309	.173	− .1824	.0255
	2.00	1.00	− .00242	.05427	.999	− .1334	.1285
		3.00	− .08090	.05540	.318	− .2146	.0528
	3.00	1.00	.07849	.04309	.173	− .0255	.1824
		2.00	.08090	.05540	.318	− .0528	.2146
Eigen centrality	1.00	2.00	− .07148	.07183	.583	− .2448	.1018
		3.00	− .18194*	.05702	.007	− .3195	− .0444
	2.00	1.00	.07148	.07183	.583	− .1018	.2448
		3.00	− .11046	.07332	.296	− .2873	.0664
	3.00	1.00	.18194*	.05702	.007	.0444	.3195
		2.00	.11046	.07332	.296	− .0664	.2873
This account follows	1.00	2.00	776.36667	475.0568	.241	− 369.7532	1922.4865
		3.00	51.09524	377.1401	.990	− 858.7912	960.9817
	2.00	1.00	− 776.36667	475.0568	.241	− 1922.4865	369.7532
		3.00	− 725.27143	484.9343	.301	− 1895.2217	444.6788
	3.00	1.00	− 51.09524	377.1401	.990	− 960.9817	858.7912
		2.00	725.27143	484.9343	.301	− 444.6788	1895.2217
Friends	1.00	2.00	159.59167	113.5047	.345	− 114.2494	433.4327
		3.00	152.12500	90.10963	.219	− 65.2731	369.5231
	2.00	1.00	− 159.59167	113.5047	.345	− 433.4327	114.2494
		3.00	− 7.46667	115.8647	.998	− 287.0015	272.0681
	3.00	1.00	− 152.12500	90.10963	.219	− 369.5231	65.2731
		2.00	7.46667	115.8647	.998	− 272.0681	287.0015
Node	1.00	2.00	396.27500*	163.6910	.049	1.3549	791.1951
		3.00	416.99405*	129.9517	.006	103.4732	730.5149
	2.00	1.00	− 396.27500*	163.6910	.049	− 791.1951	− 1.3549
		3.00	20.71905	167.0945	.992	− 382.4124	423.8505
	3.00	1.00	− 416.99405*	129.9517	.006	− 730.5149	− 103.4732
		2.00	− 20.71905	167.0945	.992	− 423.8505	382.4124
Edge	1.00	2.00	999.20000	430.8384	.062	− 40.2387	2038.6387
		3.00	970.80952*	342.0358	.017	145.6156	1796.0034
	2.00	1.00	− 999.20000	430.8384	.062	− 2038.6387	40.2387
		3.00	− 28.39048	439.7965	.998	− 1089.4414	1032.6605
	3.00	1.00	− 970.80952*	342.0358	.017	− 1796.0034	− 145.6156
		2.00	28.39048	439.7965	.998	− 1032.6605	1089.4414

*The mean difference is significant at the 0.05 level
